# Effect of pH in the Hydrothermal Preparation of Bi_2_WO_6_ Nanostructures

**DOI:** 10.3390/ma12111728

**Published:** 2019-05-28

**Authors:** Teodóra Nagyné-Kovács, Gubakhanim Shahnazarova, István Endre Lukács, Anna Szabó, Klara Hernadi, Tamás Igricz, Krisztina László, Imre M. Szilágyi, György Pokol

**Affiliations:** 1 Department of Inorganic and Analytical Chemistry, Budapest University of Technology and Economics, Műegyetem rakpart 3., H-1111 Budapest, Hungary; gubakhanim.shahnazarova@gmail.com (G.S.); imre.szilagyi@mail.bme.hu (I.M.S.); pokol.gyorgy@ttk.mta.hu (G.P.); 2 Research Institute for Technical Physics and Materials Science, Hungarian Academy of Sciences, Konkoly Thege M. út 29-33., H-1121 Budapest, Hungary; lukacs.istvan@energia.mta.hu; 3 Department of Applied and Environmental Chemistry, University of Szeged, Rerrich B.tér 1., H-6720 Szeged, Hungary; szabo.anna@chem.u-szeged.hu (A.S.); hernadi@chem.u-szeged.hu (K.H.); 4 Department of Organic Chemistry and Technology, Budapest University of Technology and Economics, Műegyetem rakpart 3., H-1111 Budapest, Hungary; igricz.tamas@oct.bme.hu; 5 Department of Physical Chemistry and Materials Science, Budapest University of Technology and Economics, Műegyetem rakpart 3., H-1111 Budapest, Hungary; klaszlo@mail.bme.hu; 6 Research Centre for Natural Sciences, Hungarian Academy of Sciences, Magyar tudósok körútja 2., H-1117 Budapest, Hungary

**Keywords:** Bi_2_WO_6_, hydrothermal synthesis, full pH range, nanostructures, morphology

## Abstract

In this study, Bi_2_WO_6_ was prepared by the hydrothermal method. The effects of reaction temperature (150/170/200 °C) and reaction time (6/12/24 h) were investigated. The role of strongly acidic pH (1 >) and the full range between 0.3 and 13.5 were studied first. Every sample was studied by XRD and SEM; furthermore, the Bi_2_WO_6_ samples prepared at different temperatures were examined in detail by EDX and TEM, as well as FT-IR, Raman and UV-vis spectroscopies. It was found that changing the temperature and time slightly influenced the crystallinity and morphology of the products. The most crystallized product formed at 200 °C, 24 h. The pure, sheet-like Bi_2_WO_6_, prepared at 200 °C, 24 h, and 0.3 pH, gradually transformed into a mixture of Bi_2_WO_6_ and Bi_3.84_W_0.16_O_6.24_ with increasing pH. The nanosheets turned into a morphology of mixed shapes in the acidic range (fibers, sheets, irregular forms), and became homogenous cube- and octahedral-like shapes in the alkaline range. Their band gaps were calculated and were found to vary between 2.66 and 2.59 eV as the temperature increased. The specific surface area measurements revealed that reducing the temperature favors the formation of a larger surface area (35.8/26/21.6 m^2^/g belonging to 150/170/200 °C, respectively).

## 1. Introduction

Metal tungstates (MWO_4_, M = Ca, Sr, Mn, Cd, Pb, Fe, Bi, etc.) belong to a family of compounds rich in potential applications, and thus are the focus of many studies. Among their valuable features, one can find favorable photocatalytic and magnetic properties [1,2,3,4,5,6,7], as well as excellent luminescent activity [8,9,10,11,12], making them a candidate for luminescence thermometry, optical heaters, and appropriate media for lasers. Furthermore, they have potential in the field of gas or humidity sensing [13,14,15,16,17,18], but can be also used as an electrode material in Li^+^/Na^+^ ion batteries, or as supercapacitors thanks to their great electrochemical properties [19,20,21,22,23,24].

Among them, Bi_2_WO_6_ is one of the most studied materials because of its remarkable photocatalytic activity and favorable band gap (approximately 2.70 eV) [25,26,27,28,29,30,31,32,33,34]. It can be synthetized in many ways such as sol-gel [25,35,36], the solvo- and hydrothermal method [33,37,38,39,40,41,42,43], spray pyrolysis [44], precipitation [1,45], or even solid-state reaction [1,46]. The most popular among them is the hydrothermal process, because of its simple apparatus and easy implementation. It also has the benefit of tailoring the crystalline structure, morphology, size of the specific surface area, and other characteristics of products through changing the synthesis parameters. Therefore, knowledge about the role of these parameters is essential. So far, numerous studies have reported on the effect of temperature, time, amount of raw materials/ additives and pH in changing the morphology (flakes, sheets, spherical figures, rods, flower or nest-like architectures, etc.) [24,43,44,45,46,47,48,49,50,51,52]. There are some studies focusing on the effect of changing the pH at 200 °C, however, in these works, the very acidic (below pH 1) and very alkaline (above pH 13) ranges were not studied. In addition, in these works, the full pH range was not investigated, but rather only a part of it, that is, between pH 1–11, 2–12, 4–8, and 4–11 [26,53,54,55,56,57,58,59].

In this report, we present the study of the effect of the full pH range (0.3–13.5) in the hydrothermal preparation of Bi_2_WO_6_. We also examined the effect of reaction temperature (150/170/200 °C) and reaction time (6/12/24 h), in order to find the most appropriate parameters for the highly crystallized products. We studied the crystalline phases and the obtained morphology of the products by XRD and SEM, respectively. Moreover, the Bi_2_WO_6_ samples prepared at different temperatures were further examined by EDX, TEM, FT-IR, Raman, and UV-vis, and their band gaps and specific surface areas were also determined.

## 2. Experimental

### 2.1. Hydrothermal Treatment

All of the chemicals were purchased from Sigma Aldrich (Darmstadt, Germany) and used without any further purifications.

For a typical synthesis, 0.49 g (0.0015 mol) Na_2_WO_4_∙2H_2_O was dissolved in 9 mL 2 M HNO_3_ (solution A) and 1.50 g (0.0030 mol) Bi(NO_3_)_3_∙5H_2_O was dissolved in 30 mL ion-exchanged water (solution B). Then, solution A was added drop by drop to solution B, which was followed by the formation of a light yellow precipitate. The pH was adjusted to the specified value by NaOH solution. After stirring for 30 minutes at 500 rpm (DLAB MS-H-PRO+) (DLAB. Instruments Ltd., Beijing, China), 30 mL of solution was transferred into a 45 mL stainless steel Teflon-lined autoclave (Parr Instruments, Moline, United States) and put into an electric furnace (Nabertherm L9/11/B410) (Nabertherm Ltd., Lilienthal, Germany) at particular temperatures and times. After the heat treatment, the autoclave was cooled down to room temperature, the solution was filtered, and the yellowish precipitate was washed several times with water and ethanol. Finally, the sample was dried in a drying oven (Memmert Ltd., Schwabach, Germany) at 60 °C for 2 h. 

The hydrothermal synthesis and their conditions are listed in Table 1.

### 2.2. Characterization 

The crystalline phases were studied by XRD (X-ray Diffraction) using a PANanalytical X’Pert Pro MPD X-ray diffractometer (Malvern Pananalytical, Almelo, The Netherlands) with Cu Kα radiation (λ = 0.15418 nm). For SEM (Scanning Electron Microscopy) and TEM (Transmission Electron Microscopy) measurements, a LEO1540 XB (LEO Electron Microscopy Inc., Thornwood, United States) and an FEI Tecnai G2 20 X-TWIN electron microscope (BIONAND, Malaga, Spain) operated at 200 keV, respectively, were used. 

For determination of the specific surface area, the as-prepared samples were evacuated at 150 °C for 24 h before the measurement. Then, low temperature N_2_ adsorption/desorption isotherms were measured at −196 °C on a Nova2000e (Quantachrome) computer-controlled apparatus (Anton Paar Ltd., Graz, Austria). The specific surface area (S*_BET_*) calculations were made using the Brunauer–Emmett–Teller (BET) model [60].

Crystallite sizes were determined using the Scherrer formula: D = kλ/(β_mcosθ), where D (Å) is the thickness of the crystallite size, k a constant (0.9), λ the wavelength of the X-ray source (1.5418 Å), β the broadening of the XRD reflection (full width at half maximum), and θ (rad) the diffraction angle. The (131) main reflection was chosen for the calculations.

For the elemental composition examinations (EDX), we applied a JEOL JSM 5500-LV instrument (Jeol Ltd., Musashino, Japan). FT-IR (Fourier-Transformation Infra Red) spectra were recorded by a Perkin Elmer 2000 FT-IR spectrometer (Perkin Elmer, Waltham, United States) between 4000 and 450 cm^−1^ applying KBr pellets (1 mg sample/ 300 mg KBr). Raman measurements were carried out by a Jobin Yvon LabRam spectrometer (Horiba, Miyanohigashi, Japan) equipped with an Olympus BX41 optical microscope using a frequency doubled Nd-YAG laser (532 nm). Diffuse reflectance UV-vis spectra were taken by a Jasco V-570 UV/VIS/NIR spectrometer (Jasco, Easton, United States).

## 3. Results and Discussion

### 3.1. Effect of Time and Temperature

**Figure 1:**
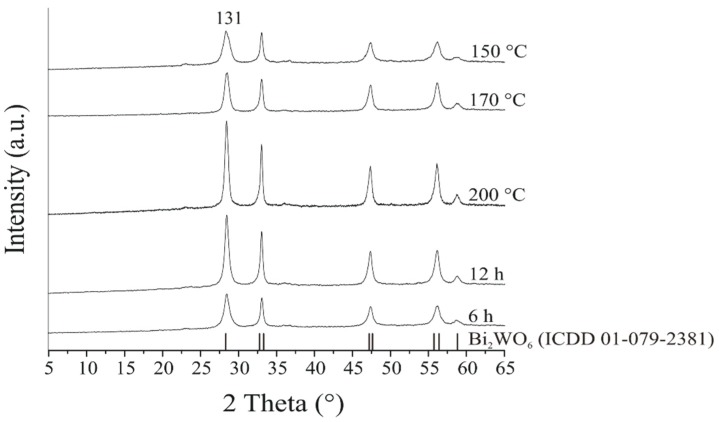
XRD patterns of the prepared Bi_2_WO_6_ samples applying different temperatures and times.

The crystalline phases of **1**–**5** were investigated by XRD (Figure 1). On the basis of the XRD patterns, **1**–**5** have five strong diffraction peaks at 28.4, 33.0, 47.3, 56.1, and 58.7°, respectively, which can be attributed to the orthorhombic Bi_2_WO_6_ phase (ICDD 01-079-2381, the main reflection (131) is labeled). Sharp, well distinguished XRD peaks indicate the well-crystallized structure without other peaks referring to impurities. Although the crystalline phases did not change, the crystallinity increased as a result of the higher reaction temperature and time, confirmed by the reflections of **3** which are the sharpest and narrowest. 

**Figure 2:**
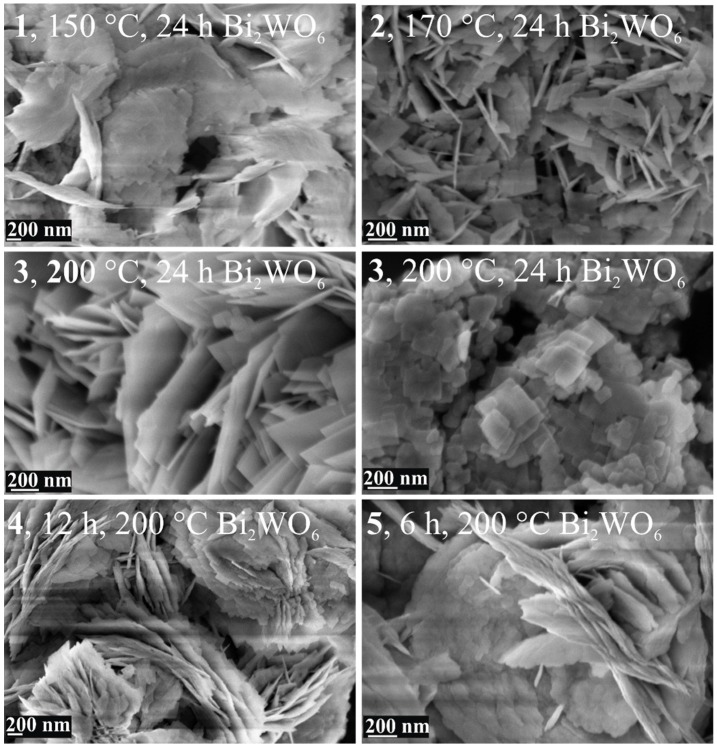
SEM images of Bi_2_WO_6_ samples prepared at different temperatures and times.

In Figure 2, the SEM images show the featuring morphology of **1**–**5**. It is clear that the sheet-like morphology is characteristic of all samples, independent of the applied reaction temperature and time. At 150 and 170 °C, the sheets are 10–20 nm thick and have various sizes, and fiber-like forms also appear (**1**–**2**, Figure 2). However, a homogenous sheet-like morphology formed at 200 °C consisting of 10–20 nm thick and 200–400 nm wide angular forms (**3**, Figure 2). Decreasing the time from 24 h to 12 and 6 h, the obtained morphology changed, because not only angular sheets, but also curved discs and fibers appear (**4**–**5**, Figure 2).

### 3.2. Effect of pH

When changing the pH, both the crystalline phases and the morphology go through significant transformations.

**Figure 3:**
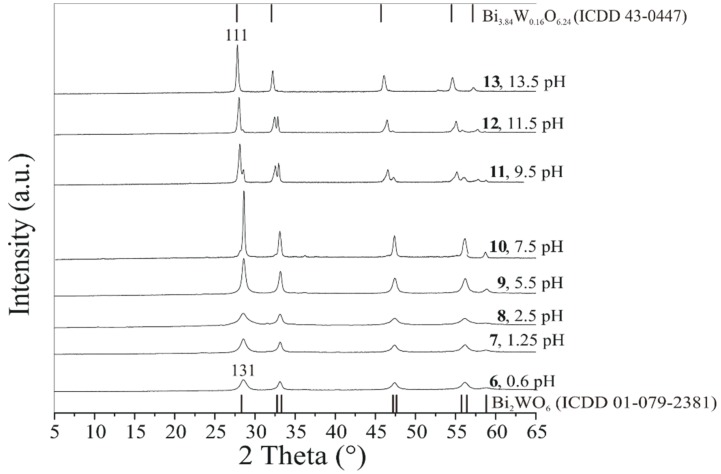
XRD patterns of the Bi_2_WO_6_ samples prepared at 200 °C, 24 h using different pH values.

On the basis of the XRD patterns of **6**–**12**, in the acidic range from 0.6 to 5.5 pH, all samples were identified as pure orthorhombic Bi_2_WO_6_ (ICDD 01-079-2383, the main reflection (131) is labeled, Figure 3). In the alkaline pH range, from a pH value of 7.5 to 13.5, the crystalline phases of the samples turned to a mixture of Bi_2_WO_6_ and Bi_3.84_W_0.16_O_6.24_ (ICDD 43-0447, **10**–**13**, the main peak (111) is labeled, Figure 3). It can clearly be seen that the XRD peaks of the Bi_3.84_W_0.16_O_6.24_ phase become gradually stronger along with the increasing pH, and finally develop into the most significant reflections. 

The distinct, sharp XRD peaks of the samples prepared in the alkaline range (**10**–**13**) indicate well-crystallized materials, in contrast with **6**–**9,** which were synthetized using acidic pH (Figure 3).

**Figure 4:**
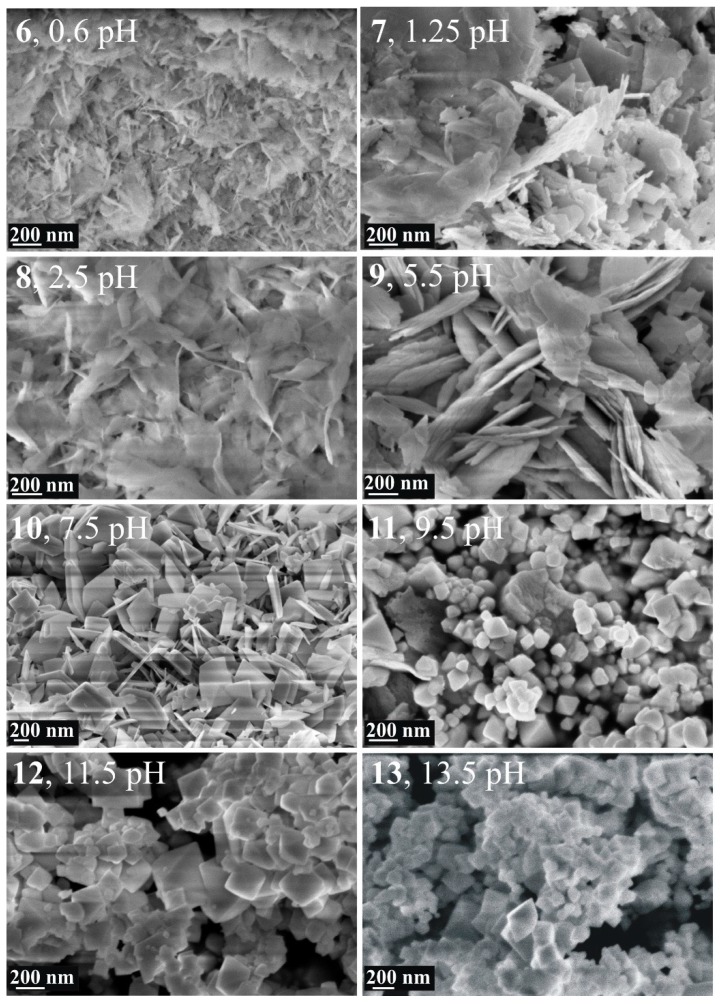
SEM images of Bi_2_WO_6_ samples synthetized at 200 °C, 24 h using different pH values.

Significant changes were also observed in the obtained morphology of **6**–**13** (Figure 4). In the case of **6**, 100–200 nm long fibers and other irregular shapes formed, while the morphology of **7** and **8** is composed of various sheet-like forms, together with irregular, curved figures of different sizes. In **9**, however, the Bi_2_WO_6_ phase appeared in the form of only sheets with 10–20 nm thickness. This morphology is similar to **3**, where the Bi_2_WO_6_ phase was also obtained with a nanosheet morphology. Here, in the case of **9**, however, the appearance of sheets is not so uniform, and the sheets have mostly curved edges, not strictly angular.

Further increasing the pH, it was found that the sheet-like morphology gradually transformed into small cubic and octahedral shapes. In the case of **10**, where the Bi_3.84_W_0.16_O_6.24_ phase also appears beside Bi_2_WO_6_, the morphology becomes a mixture of thin, only 10–50 nm and larger, even 100–300 nm thick sheets (**10**, Figure 4). As the ratio of crystalline Bi_3.84_W_0.16_O_6.24_ phase increases in **11**–**13**, the crystalline appearance turns into a cube and octahedral-like morphology (Figure 4). In **11**, these forms are uniform in size, between 100–200 nm, but in **12** and **13**, where the ratio of the Bi_3.84_W_0.16_O_6.24_ phase is much stronger than Bi_2_WO_6_, larger figures, with 200–300 nm edges, can be observed as well.

It is known that at low pH values, the hydrolysis of Bi^3+^ is restrained because of the great amount of H^+^ ions, and thus the nucleation rate of Bi_2_WO_6_ is favored against crystal growth. This indicates the formation of many nanometer-sized Bi_2_WO_6_ nuclei whose sheet-like formation is derived from the intrinsic anisotropic layered structure. In the alkaline range, however, the facile hydrolysis of Bi^3+^ ions favors the crystal growth through precipitation with the also soluble WO_4_^2-^ anion, resulting not only in a new phase (Bi_3.84_W_0.16_O_6.24_), but in cubes and octahedral shapes as well (Figure 4) [32,41,47,49,61,62,63].

### 3.3. Further Characterization of Samples Synthesized at Various Temperatures

Further investigations were carried out using the pure Bi_2_WO_6_ samples (**1**–**3**), which were prepared at 150, 170, and 200 °C, 24 h.

#### 3.3.1. TEM, Specific Surface Area, Crystallite Size, and EDX

**Figure 5:**
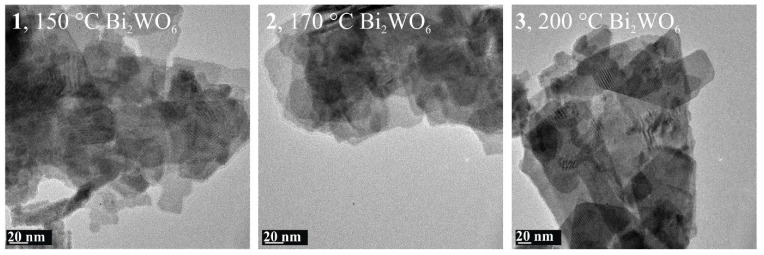
TEM images of the Bi_2_WO_6_ synthetized at different temperatures.

To further investigate the morphology of **1**–**3**, TEM images were taken (Figure 5). In every image, highly crystallized Bi_2_WO_6_ can be seen, which is self-assembled by many strongly agglomerated nanosheets in good accordance with the SEM images. 

A comparison of the surface area of the samples obtained at different temperatures revealed that the largest area, 35.8 m^2^/g, belonged to **1**, that is, to the sample prepared at 150 °C. **2** and **3**, prepared at 170 and 200 °C, respectively, had a smaller surface (26 and 21.9 m^2^/g) because of the larger crystallite size developing at higher temperatures. This effect is well-known in the literature and has been already reported many times, as well as in the case of other materials. The higher temperature strongly influences the crystal growth, providing sufficient energy for smaller grains to grow and form bigger crystallites, while the specific surface area reduces [27,48,64,65,66]. This phenomenon corresponds with the calculated crystallite size of the samples, which are 16, 19.7, and 35for the sample prepared at 150, 170, and 200 °C, respectively.

The EDX results (Table 2) show that only Bi, W, and O can be found in the samples with atomic percentages close to the expected values (EDX has ± 5%–10% relative error, which can be even higher in the case of elements with a lower atomic number).

**Figure 6:**
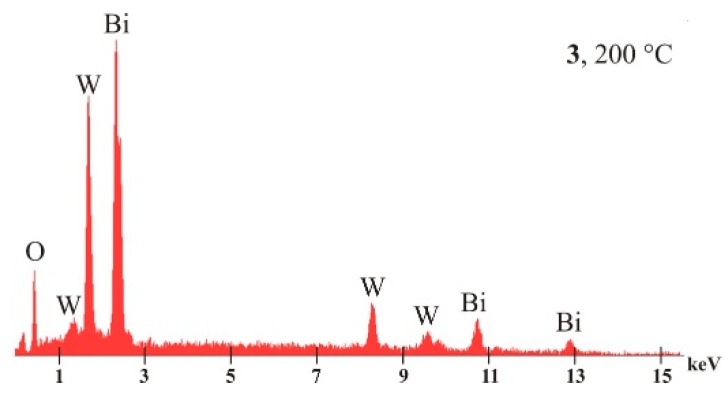
A typical EDX spectrum of the Bi_2_WO_6_ samples prepared at 150, 170, and 200 °C, 24 h.

A typical EDX spectrum can be seen in Figure 6. On the spectrum, only the signs of the main components appear (O, W, and Bi), indicating that no other elements referring to other phases or impurities are present. 

#### 3.3.2. FT-IR, Raman, and UV-vis Spectroscopy Results

**Figure 7:**
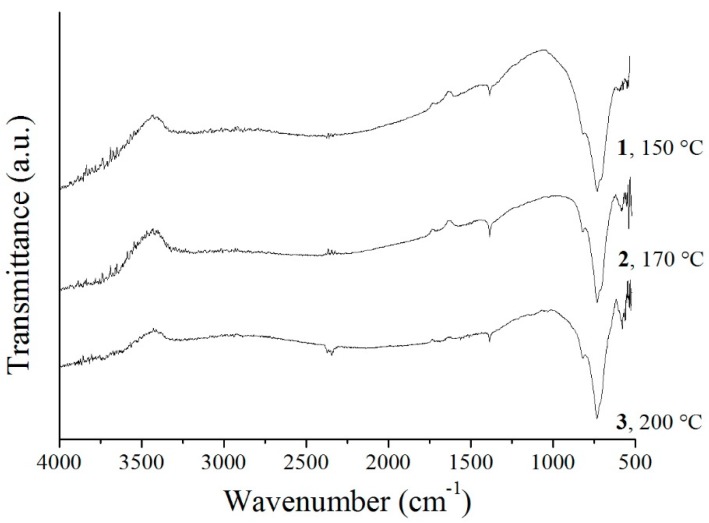
FT-IR spectra of the Bi_2_WO_6_ samples prepared at different temperatures.

In the FT-IR spectra of **1**–**3**, between 500 and 1000 cm^−1^, the characteristic peaks of W–O modes can be observed (Figure 7). The band at 820 cm^−1^ belongs to the stretching vibration of Bi–O, and the others below 750 cm^−1^ are assigned to the stretching and bridging stretching mode of W–O and W–O–W, respectively [25,28,50,54,67].

**Figure 8:**
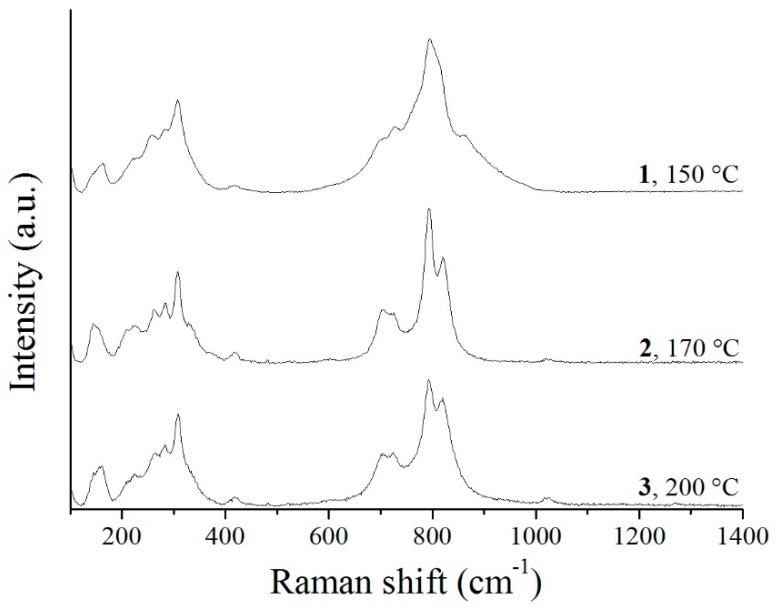
Raman spectra of the Bi_2_WO_6_ samples synthetized at different temperatures.

Raman spectra show well distinct peaks revealing the Bi_2_WO_6_ structure (Figure 8). The double peak at 800 cm^−1^ belongs to the antisymmetric and symmetric A_g_ modes of terminal O–W–O vibration. The band at 710 cm^−1^ can be ascribed to the antisymmetric bridging mode originated in the tungstate chain. The weak band appearing at 433 cm^−1^ is assigned to the antisymmetric mode of WO_6_ octahedral, while at 310 cm^−1^, the translational mode of the simultaneous move of Bi^3+^ and WO_6_^6−^ can be found. The band at 300 cm^−1^ corresponds to the mode of the WO_2_ terminal groups (Figure 1) [29,42,50,68,69].

**Figure 9:**
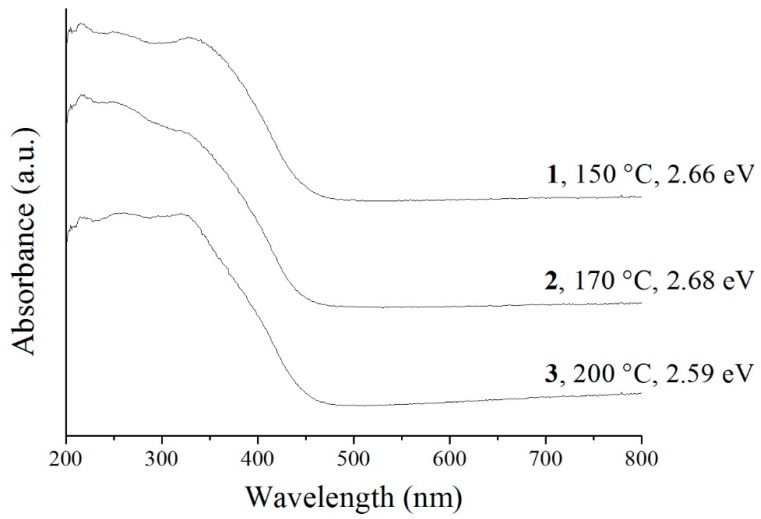
UV-vis spectra and the calculated band gap of the Bi_2_WO_6_ samples made at different temperatures.

The diffuse reflectance UV-vis spectra of **1**–**3** reveal a definite absorption edge at 480–490 nm in the case of all samples (Figure 9). **1**–**3** have visible light absorption ability as well, in good agreement with their light yellow color.

Their band gaps were also calculated using the equation $\alpha h\upsilon =A\;{\left(h\upsilon -E_{g}\right)}^{n}$, where *α*, *hυ*, *A*, and *E_g_* refer to molar absorption coefficient, photon energy, general constant, and band gap energy, respectively. *N* depends on the direct or indirect allowed or forbidden type of the electron transition of the material, and is 2 in the case of Bi_2_WO_6_, which is an indirect semiconductor. Plotting αhυ against hυ (Tauc-plot), then drawing a tangent line onto the linear range and extrapolating, the band gap energy can be determined. To approximate *A*, the Kubelka–Munk function was used. The calculated band gaps are 2.66 and 2.68 eV in the case of **1** and **2**, respectively, but for **3**, it is only 2.59 eV (Figure 9). These values are otherwise in good agreement with the values reported in the literature (2.59 and 2.81 eV) [28,31,33,34,70], and make the prepared Bi_2_WO_6_ samples promising candidates in the field of photocatalysis. They show a slight temperature-dependent tendency, as the smallest band gap belongs to **3**, which was prepared at the highest temperature, and thus has the most ordered structure, while **1** and **2** are more similar to each other regarding the degree of crystallinity and atomic order, and thus have a similar band gap value.

## 4. Conclusion

In this report, we successfully investigated the effect of reaction temperature (150/170/200 °C), reaction time (6/12/24 h), and pH (0.3/0.6/1.25/2.5/5.5/7.5/9.5/11.5/13.5) on the obtained morphology and crystalline phases in the hydrothermal preparation of Bi_2_WO_6_. Our aim was to study the full pH range using very acidic (below pH 1) as well as very alkaline (above pH 13) ranges, because the effect of these has not been examined yet. Pure, crystalline Bi_2_WO_6_ formed independently of the used temperature and time at pH 0.3, but the crystallinity varied. It was enhanced as the time and temperature increased. The morphology was, however, a mixture of 10–20 nm thick sheets and fibers at 150 and 170 °C, 24 h, while it was consisted of uniform, angular nanosheets when the temperature was 200 °C and 24 h was used. When the time was decreased to 12 and 6 h, the obtained morphology contained sheets and other forms (discs, fibers) as well. Varying the pH resulted in significant changes both in the crystalline phases and in the morphology. In the acidic range (from 0.6 to 5.5), all samples were pure Bi_2_WO_6_. At 0.6 pH, the morphology emerged in irregular forms, while at 1.25 and 2.5 pH, it was a mixture of sheets and other irregular figures. When the pH was set to 5.5, however, Bi_2_WO_6_ was obtained in the form of nanosheets. Further increasing the pH, at values of 7.5/9.5/11.5 and 13.5, the samples contained Bi_2_WO_6_ and Bi_3.84_W_0.16_O_6.24_ phases, and the structure and morphology gradually transformed into cube- and octahedral-like forms of the new phase. The pure Bi_2_WO_6_ samples prepared at 150/170/200 °C were studied in detail. EDX, TEM, as well as FT-IR, Raman, and UV-vis spectroscopies revealed their elemental composition, sheet-like structure, and optical properties, respectively. Their band gaps were calculated and it was found that they varied between 2.66 and 2.59 eV as the temperature increased. A comparison of the specific surface areas and crystallite sizes showed that the larger the area, the lower the temperature (35.8/26/21.6 m^2^/g belonging to samples prepared at 150/170/200 °C, respectively). 

## Figures and Tables

**Table 1:** Summary of the carried out hydrothermal reactions.

Sample	Temperature (°C)	Time (h)	pH	Crystalline Phase(s)
**1**	150	24	0.3	Bi_2_WO_6_
**2**	170	24	0.3	Bi_2_WO_6_
**3**	200	24	0.3	Bi_2_WO_6_
**4**	200	12	0.3	Bi_2_WO_6_
**5**	200	6	0.3	Bi_2_WO_6_
**6**	200	24	0.6	Bi_2_WO_6_
**7**	200	24	1.25	Bi_2_WO_6_
**8**	200	24	2.5	Bi_2_WO_6_
**9**	200	24	5.5	Bi_2_WO_6_
**10**	200	24	7.5	Bi_2_WO_6_, Bi_3.84_W_0.16_O_6.24_
**11**	200	24	9.5	Bi_2_WO_6_, Bi_3.84_W_0.16_O_6.24_
**12**	200	24	11.5	Bi_2_WO_6_, Bi_3.84_W_0.16_O_6.24_
**13**	200	24	13.5	Bi_2_WO_6_, Bi_3.84_W_0.16_O_6.24_

**Table 2:** Crystallite size, specific surface area, and EDX results of **1**–**3**.

		1, 150 °C	2, 170 °C	3, 200 °C
S_BET_ (m^2^/g)		35.8	26	21.9
Crystallite size (nm)		16.0	19.7	35.0
EDX (atom%)	Bi	25.3	26.8	28.3
	*W*	14.9	13.2	17.1
	*O*	59.8	61.0	60.3
